# The Molecule Role Ontology: An Ontology for Annotation of Signal Transduction Pathway Molecules in the Scientific Literature

**DOI:** 10.1002/cfg.432

**Published:** 2004

**Authors:** Satoko Yamamoto, Takao Asanuma, Toshihisa Takagi, Ken Ichiro Fukuda

**Affiliations:** 1 Institute for Bioinformatics Research and Development Japan Science and Technology Agency Tokyo Japan; 2 Graduate School of Frontier Sciences University of Tokyo Chiba Japan; 3 Computational Biology Research Center National Institute of Advanced Industrial Science and Technology 2–43 Aomi Koutou-ku Tokyo 135-0064 Japan

## Abstract

In general, it is not easy to specify a single sequence identity for each molecule name that appears in a pathway in the scientific literature. A molecule name may stand for concepts of various granularities, from concrete objects such as H-Ras and ERK1 to abstract concepts or categories such as Ras and MAPK. Typically, the relations among molecule names derive a hierarchical structure; without a proper way to handle this knowledge, it becomes ever more difficult to develop a reliable pathway database. This paper describes an ontology that is designed to annotate molecules in the scientific literature on signal transduction pathways.


**Comparative and Functional Genomics**
*Comp Funct Genom* 2004; **5**: 528–536.
Published online in Wiley InterScience (www.interscience.wiley.com). **DOI:** 10.1002/cfg.432

Conference Paper

### The Molecule Role Ontology: an ontology for annotation of signal transduction pathway molecules in the scientific literature

Satoko Yamamoto[1], Takao Asanuma[1], Toshihisa Takagi[2] and Ken Ichiro Fukuda[3]*

[1] *Institute for Bioinformatics Research and Development, Japan Science and Technology Agency, Tokyo, Japan*
[2] *Graduate School of Frontier Sciences, University of Tokyo, Chiba, Japan*
[3] *Computational Biology Research Center, National Institute of Advanced Industrial Science and Technology, Tokyo, Japan*

**Correspondence to:
Ken Ichiro Fukuda,
Computational Biology Research Center, National Institute of Advanced Industrial Science and Technology, 2–43 Aomi, Koutou-ku, Tokyo 135-0064, Japan.
E-mail: fukuda-cbrc@aist.go.jp*

Received: 5 October 2004
Revised: 20 October 2004
Accepted: 22 October 2004

### Abstract

**In general, it is not easy to specify a single sequence identity for each molecule name that appears in a pathway in the scientific literature. A molecule name may stand for concepts of various granularities, from concrete objects such as H-Ras and ERK1 to abstract concepts or categories such as Ras and MAPK. Typically, the relations among molecule names derive a hierarchical structure; without a proper way to handle this knowledge, it becomes ever more difficult to develop a reliable pathway database. This paper describes an ontology that is designed to annotate molecules in the scientific literature on signal transduction pathways. Copyright © 2004 John Wiley & Sons, Ltd.**

**Keywords:** ontology; signal transduction; text mining; scientific literature; pathway database; annotation; manual curation; molecule name; protein name dictionary

### Background

As the genomes of many species have now been sequenced, the target of biological knowledge acquisition has shifted from elucidating the features of genes and proteins to discovering combinations of genes and proteins, and of their interactions, that constitute biological functions, i.e. pathways. The integration of biological data from different sources by cross-referencing has progressed significantly. However, pathway data (e.g. disease pathways) or descriptions of the molecular mechanisms underlying biological processes continue to reside primarily in the scientific literature or in the comment descriptions of databases such as Swiss-Prot (Boeckmann *et al*., 2003) and InterPro (Mulder *et al*., 2003), in the form of free text or diagrams.

Pathway data are ‘processed’ rather than ‘raw’ knowledge or data, and are integrated from multiple knowledge sources. Signal transduction pathway data are good examples of this type of knowledge. Their constituent biological entities are highly diverse and range from metal ions to proteins to biological processes in general. Likewise, the kinds of interactions that connect biological entities are diverse. Therefore, the type of knowledge that we want to represent in a pathway database and the type of query that we want to conduct in a pathway database are not apparent. Consequently, there are many ongoing pathway database projects, each of which targets different types and levels of knowledge. Historically, pathway database development started with metabolic pathways, as these could be seen as the most concrete description of biological processes. Subsequently, several databases attempted to encode other levels of biological processes, such as gene regulation, signal transduction, and disease pathways, with the ultimate aim being the complete description of biological processes found in the literature. This requires wider coverage of concepts than is required for biochemical (metabolic) pathways.

Copyright © 2004 John Wiley & Sons, Ltd.

INOH (Integrating Network Objects with Hierarchies; INOH database website), PATIKA (Demir *et al.*, 2004) and Reactome (Joshi-Tope *et al.*, 2003) focus on biological processes at various levels, with INOH and Reactome including manual curation from the scientific literature. These databases are process-orientated, in the sense that they use a compound-graph structure (Fukuda and Takagi, 2001) and are able to annotate sub-pathways or sub-processes.

A classical method of developing a signal transduction pathway database is to represent pathways by a set of binary (and sometimes trinal) relations; examples are CSNDB (Takai-Igarashi and Kaminuma, 1999) and TRANSPATH (Krull *et al.*, 2003). However, as a pathway is a connected component of the set of binary relations, it is difficult to annotate sub-pathways with their functions. BioCarta (BioCarta website) is a clickable-map based database. Signal Transduction Knowledge Environment (STKE; STKE website) and the Alliance for Cellular Signaling (AfCS; AfCS website) provide integrated knowledge portals with pathway maps and review documents. There are several databases that focus on protein–protein interaction data, including BIND (Bader *et al.*, 2003) and DIP (Salwinski *et al.*, 2004). The most mature pathway databases, i.e. metabolic pathway databases, EcoCyc (Karp *et al.*, 2004) and KEGG (Kanehisa and Goto, 2000), have been extended to handle gene regulation data. Also worth mentioning is BioPAX (BioPAX website), which is not a database but a data exchange format for pathway data. Its current level 1 release is limited to the exchange of metabolic pathway data. The roadmap includes gene regulation (level 3) and abstract set relationships (level 4). Since BioPAX is a data exchange format, its ontology does not provide any pathway information *per se*, rather, it defines what types of attributes a reaction should have.

In all of these cases, the curator has to annotate meanings to each object that appears in the pathway data. To accomplish this, the INOH project provides a set of ontologies for pathway annotation. Each of our ontologies annotates certain types of objects or attributes of objects in a pathway, such as molecules, phenotypes, localization and species. This paper describes an ontology that is designed to annotate molecules in the scientific literature on signal transduction pathways. We first discuss the features of molecule names in biomedical texts and the problems caused by these features. Then we describe our ontology development process and demonstrate the application of our Molecule Role Ontology in the INOH database. We then introduce other approaches related to the Molecule Role Ontology and discuss future directions for our work.

### Features of molecule names in the scientific literature

In general, it is not easy to specify a single sequence identity for each molecule name that appears in a pathway in the scientific literature. A molecule name may stand for concepts of various granularities, from concrete objects, such as H-Ras and ERK1, to abstract concepts or categories, such as Ras and MAPK. Figure 1 is a typical example of a pathway description (MAPK pathway) found in the literature. Looking at the figure, a biologist with proper background knowledge understands that ‘tyrosine kinase receptor’ stands for receptors such as the EGF-, PDGF- and insulin receptors (Widmann *et al.*, 1999), that ‘PI3K’ represents a complex that consists of a p110 catalytic subunit and a p85 regulatory subunit, that ‘Ras’ refers to H-Ras, K-Ras4A, K-Ras4B, and N-Ras, that ‘Raf’ can mean c-Raf-1, A-Raf, and B-Raf, and that ‘MEK’ stands for MEK1 or MEK2, and ‘ERK’ for ERK1 or ERK2 (Shields *et al.*, 2000). On the

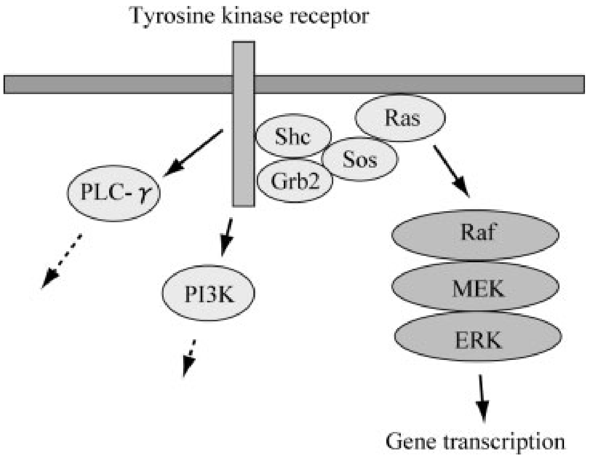

**Figure 1.** A typical pathway description found in the scientific literature (MAPK pathway). It shows how signals activate MAP kinase through tyrosine kinase receptor and adapter proteins

Copyright © 2004 John Wiley & Sons, Ltd. 

other hand, for Grb2, if the pathway is a human pathway, then its sequence can be identified by the UniProt (Apweiler *et al.*, 2004) Accession No., in this case P29354.

As current pathway descriptions in the scientific literature contain molecule names that vary in their granularity, we prepared the following categories:

- *Concrete-Names*: names specific enough to identify each of their sequences (Grb2).
- *Abstract-Names*: names that stand for several sequences (Ras, Raf, MEK, ERK).
- *Complex-names*: names that refer to complexes (PI3K).
- *Function-Names*: names that describe only functions without specifying a concrete or abstract molecule name (e.g. tyrosine kinase receptor).

Typically, the relations among the molecule names result in a hierarchical structure. Molecular biologists usually classify proteins according to their functions and, although some are classified according to their sequence similarities, not all categories are based on sequences. Rather, some classifications are based on molecular functions in the context of protein interactions and signal transduction, e.g. InterPro defines the Smad (Dwarfin) protein family as a single family with three domains, the Dwarfin protein, Dwarfin protein A, and SMAD/FHA. However, in the scientific literature, molecular biologists subdivide the Smad protein category into three Abstract-Names, R-Smad, I-Smad and Co-Smad.

### The molecule name problem in pathway databases

Although biologists know that H-Ras is one of the Ras's, and ERK1 is one of the MAPKs, a computer has no such background knowledge. This presents a problem in the development of a literature knowledge-based database, since by encoding only the names of molecules, the relationship between H-Ras and Ras is lost. A simple synonym dictionary would be insufficient to represent the relations between names. For example, to what does 'NF-$\kappa$B' in the literature refer? Mammals express five Rel (NF-$\kappa$B) family proteins that act as various homo- and heterodimers (Karin and Lin, 2002). Such information cannot be represented completely with synonyms. To perform a complicated pathway query that must traverse the concept space of abstract and concrete molecule names, the relationships between abstract names and concrete substances must be managed unitarily, and consistently.

Typically, however, this information on molecular functions or families is encoded directly in the form of free text or hyperlinks in pathway databases. For example, the 'Ras' link in a clickable map may take the user to molecule information on 'H-Ras', but some 'Ras' may not have links to 'H-Ras' simply by mistake. The drawback of this approach is the difficulty of managing the data in a unitary way while retaining integrity. Actually, in the above example, 'Ras' has four types of mammalian proteins, i.e. H-Ras, N-Ras, K-Ras4A, and K-Ras4B. Without a proper way of handling this knowledge, it becomes very difficult to develop a reliable pathway database, especially if it is developed by distributed co-curation processes. The fundamental problem is the hard encoding of background knowledge about molecules into database links. To resolve this problem, we propose the use of an ontology that represents background knowledge. Annotation of objects with this ontology renders computers capable of computing pathway knowledge.

### Methods

The Molecule Role Ontology is a reusable and explicit classification system of molecules that enables a complicated search on a literature-based knowledge base. The ontology was developed using DAG-Edit (Figure 2; DAG-Edit website), by the following procedure.

Higher-level terms of the ontology correspond to Function-Names. The top concept for proteins is the term 'protein'. Terms referring to these Function-Names were extracted manually from reviews and original articles on molecular biology. All such terms were arranged in a hierarchical structure. The hierarchy was designed carefully, based on descriptions in the literature, life science dictionaries and text books, and annotations of protein databases such as SwissProt, so that the classification is general and acceptable to biologists. This classification is a conceptual classification of molecular functions in protein interactions

Copyright © 2004 John Wiley & Sons, Ltd. 

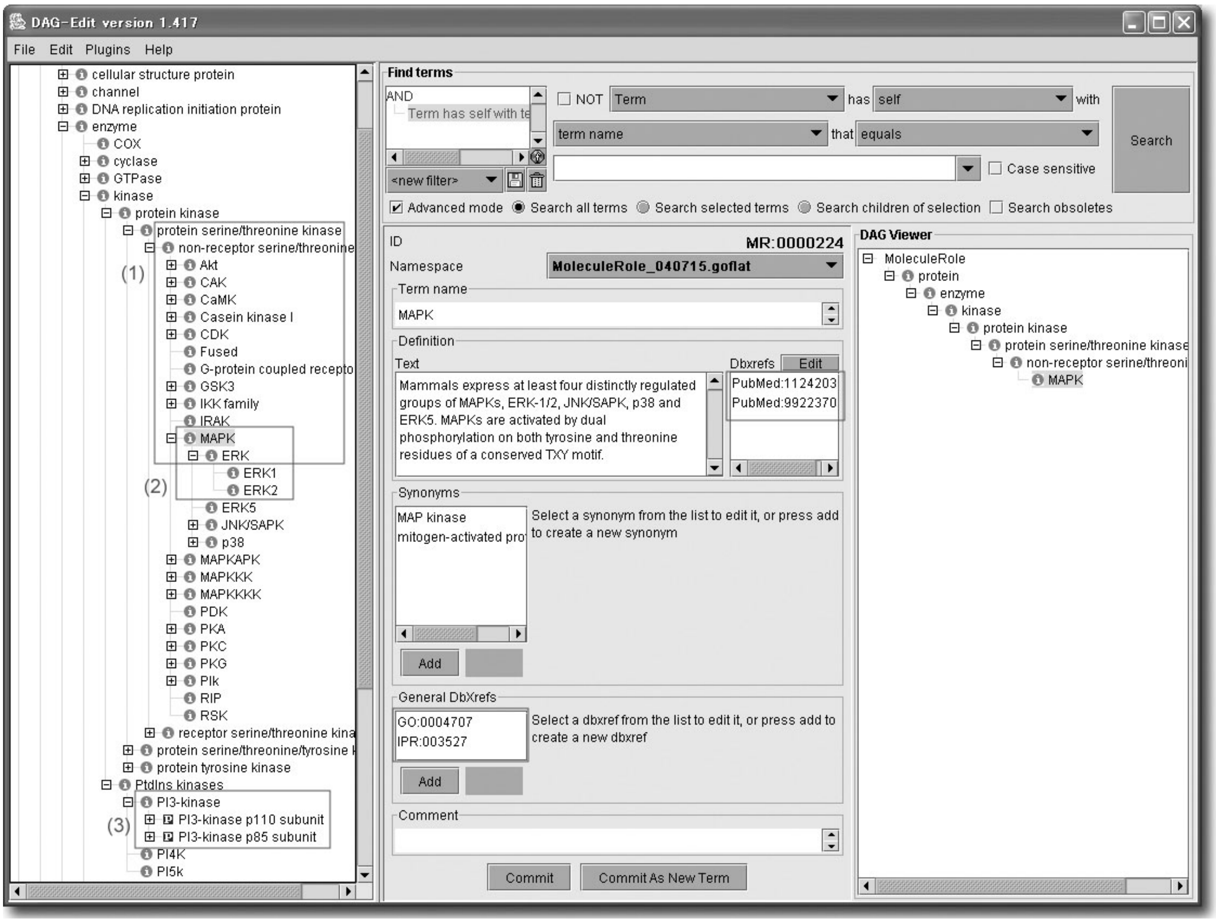

**Figure 2.** A DAG-Edit screenshot of the ontology . (1) 'is-a' relations between Function-Names and Abstract-Names (e.g. protein serine/threonine kinase and MAPK), (2) 'is-a' relations between Abstract-Names and Concrete-Names (e.g. MAPK and ERK1). (3) 'part-of' relations between a complex and its subunit (e.g. PI3-kinase and PI3-kinase p110 subunit). PubMed links as evidence for the classification and general links to GO, InterPro, UniProt, MeSH and KEGG COMPOUND are boxed and signal transduction and is not identical to families derived from sequence analysis. For example, it contains concepts such as 'adapter protein', referring to molecules that mediate molecular interactions, which are used as branch points of a signal, and 'signal regulator', which refers to molecules that set a signal to be positive or negative. Evidence for the design of the hierarchy is stored in the DAG-Edit 'Definition Text' field and the links to the original articles or reviews are stored in the 'Definition DbXrefs' field. Where possible, each higher-level concept in the ontology has a 'General DbXrefs' link to a corresponding term in the Gene Ontology (GO; The Gene Ontology Consortium, 2004).

Leaf nodes in the ontology correspond to Concrete-Names. For each of these terms we manually identified the UniProt (SwissProt/TrEMBL) Accession No. and stored the information in 'General DBXrefs'. Molecular complexes were also added to the ontology, and the relations between a complex and its subunits were defined as 'part-of' relationships. Chemical compounds that are particularly important in the signal transduction field (e.g. second messengers, such as cAMP and the calcium ion) were classified and added to the ontology. The top concept for chemical compounds is the term 'chemical'. Each leaf node in the chemical hierarchy has a KEGG COMPOUND database link in the 'General DbXrefs' field. For each higher-level term

Copyright © 2004 John Wiley & Sons, Ltd. 

in the chemical tree, a MeSH Unique ID (MeSH website) was annotated in the 'General DbXrefs' field.

### The Molecule Role Ontology and its applications

The Molecule Role Ontology encodes: (1) relations between Function-Names and Abstract-Names (e.g. protein serine/threonine kinase and MAPK); (2) relations between Abstract-Names and Concrete-Names (e.g. MAPK and ERK1); (3) complexes and their subunits (e.g. PI3-kinase and PI3-kinase p110 subunit); (4) Concrete-Names and UniProt Accession Nos (Figure 2). The hierarchies of (1) and (2) are defined by an 'is-a' relation, while the hierarchy of (3) is defined as a 'part-of' relation. As stated above, the Molecule Role Ontology is divided into two classes, 'chemical' and 'protein', at the top. Each leaf entry in the protein tree has UniProt links for human, mouse, and rat. In this sense, a leaf node is still a kind of class and the links to a UniProt entry represent a kind of 'instance-of' relation. Entries other than leaf entries (nodes) have links to GO terms. Chemical leaf nodes have links to KEGG, and chemical internal nodes have links to MeSH terms. The numbers of leaf nodes, internal nodes, and links for proteins and chemicals are shown in Table 1.

The Molecule Role Ontology can be accessed through our Ontology Viewer web application (**http://www.inoh.org/ontology-viewer**). The Ontology Viewer allows the user to search the ontology by names, synonyms, and ontology IDs (Figure 3). By clicking a search result, the user can see where the term is located in the ontology hierarchy. By clicking the term in the hierarchy, a new window appears in which the user can browse the value of each attribute, such as UniProt IDs, KEGG IDs, GO IDs, and synonyms. The graph representation below the attribute values displays the parent and child concepts. By clicking a parent or child node, another new window appears that shows that node in the centre.

**Table 1.** Molecule role ontology data (version 1.03–28 September 2004)

| | |
|---|---|
| Molecule role concepts, total no. | 1927 |
| Protein concepts, total no. | 1870 |
| No. of nodes linked to GO, InterPro/no. of nodes (protein) | 188/446 |
| No. of leaves linked to UniProt/no. of leaves (protein) | 1287/1,424 |
| Total no. of UniProt links | 3613 |
| Chemical Concepts, total no. | 56 |
| No. of nodes linked to MeSH/no. of nodes (chemical) | 6/18 |
| No. of leaves linked to KEGG/no of leaves (chemical) | 38/38 |

The Molecule Role Ontology was developed as part of our ongoing pathway database project INOH. It is one of the ontologies used to annotate objects in our pathway data. The Molecule Role Ontology defines the meaning of Protein objects, Chemical Substance objects and Molecular Complex objects. INOH pathway data can be queried via our web-application front-end FREX (Fukuda *et al.*, 2003). FREX allows the user to search and browse pathway data. Two of the unique features of FREX are its support of compound graph-based pathway data with ontological annotations and ontology-based 'query relaxation' searching. Figure 4 shows an example query that utilizes the Molecule Role Ontology. In Figure 4A, the user enters a keyword, 'ubiquitin ligase', and specifies his/her intention about the keyword by specifying an ontology in the left pull-down menu 'Fields' (in this case, the Molecule Role Ontology). In the right pull-down menu 'Query Relaxation', the user specifies how much to traverse the ontology tree upwards or downwards. The result of the search is a list of pathways, as shown in Figure 4B. By selecting pathways from the list, the user can browse them in a Java applet (Figure 4C). The highlighted node in Figure 4C indicates that the user got a hit to 'Smurf'. The reason for the Smurf hit from the keyword 'ubiquitin ligase' is that the Molecule Role Ontology says Smurf is a ubiquitin ligase (Figure 4E). Conducting this type of query by a simple keyword search without ontological annotation would be difficult because the system does not know that the character string 'ubiquitin ligase' is related to the string 'Smurf'.

### Some other approaches

GO's Molecular Function defines relations between concepts of molecular functions; abstract molecule names such as ERK are not included. Likewise, the relations between molecular functions and concrete molecules (genes) are not included in the ontology. Instead, they are encoded as annotations to molecules in protein and gene databases.

Copyright © 2004 John Wiley & Sons, Ltd.

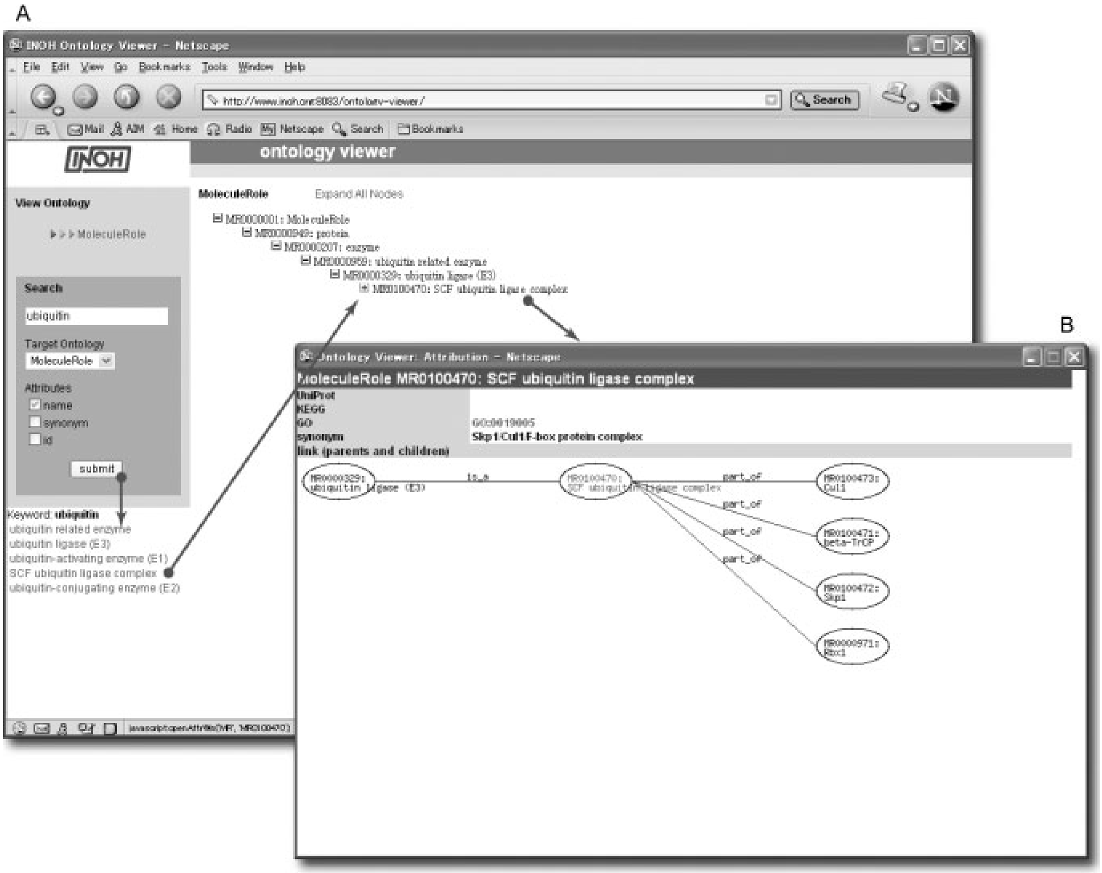

**Figure 3.** (A) INOH Ontology Viewer top page. From 'View Ontology', select an ontology to browse (currently only MoleculeRole Ontology is available). To conduct a search: (1) enter a keyword and select a 'Target Ontology', select attributes; (2) select the term to be browsed and the tree structure will be displayed; (3) select a term from the tree and click. A new window that shows the attribute values of the term will appear. (B) Ontology Viewer attribute value window. It shows UniProt, KEGG, GO IDs and synonyms, the selected term, and its parent and child concepts

This is quite reasonable and natural, since GO is designed for gene annotation. On the other hand, the Molecule Role Ontology is designed to annotate molecule objects in pathways, and therefore includes Function-Names, Abstract-Names and Concrete-Names.

Protein family databases define families according to their common domains or structures. As stated above, InterPro has a single Dwarfin (Smad) protein family. However, in the literature it is subdivided into three classes, according to their roles. Another example that highlights the difference between protein family databases and the Molecule Role Ontology is protein tyrosine kinase. While protein tyrosine kinase has several sub-families in the literature, such as the JAK family, Src family and BTK family, the only family registered in InterPro is JAK. Src kinase is not registered as a family but is registered by its domains, e.g. the SH2 domain.

TRANSPATH has a hierarchy of molecules. It consists of several types such as 'family', 'orthologue', and 'basic'. The meaning of 'family' is close to that of Function-Name in the Molecule Role Ontology, 'orthologue' corresponds to leaf nodes in the Molecule Role Ontology, and 'basic' is an entity that has a unique sequence identity and corresponds to the UniProt link in leaf nodes of the Molecule Role Ontology. However, the meaning of the relations between these types is not defined explicitly.

Reactome defines 'PhysicalEntity' by 'GenericEntity', 'ConcreteEntity', 'Complex', and 'SimpleEntity'. Using these classes, it tries to capture

Copyright © 2004 John Wiley & Sons, Ltd. 

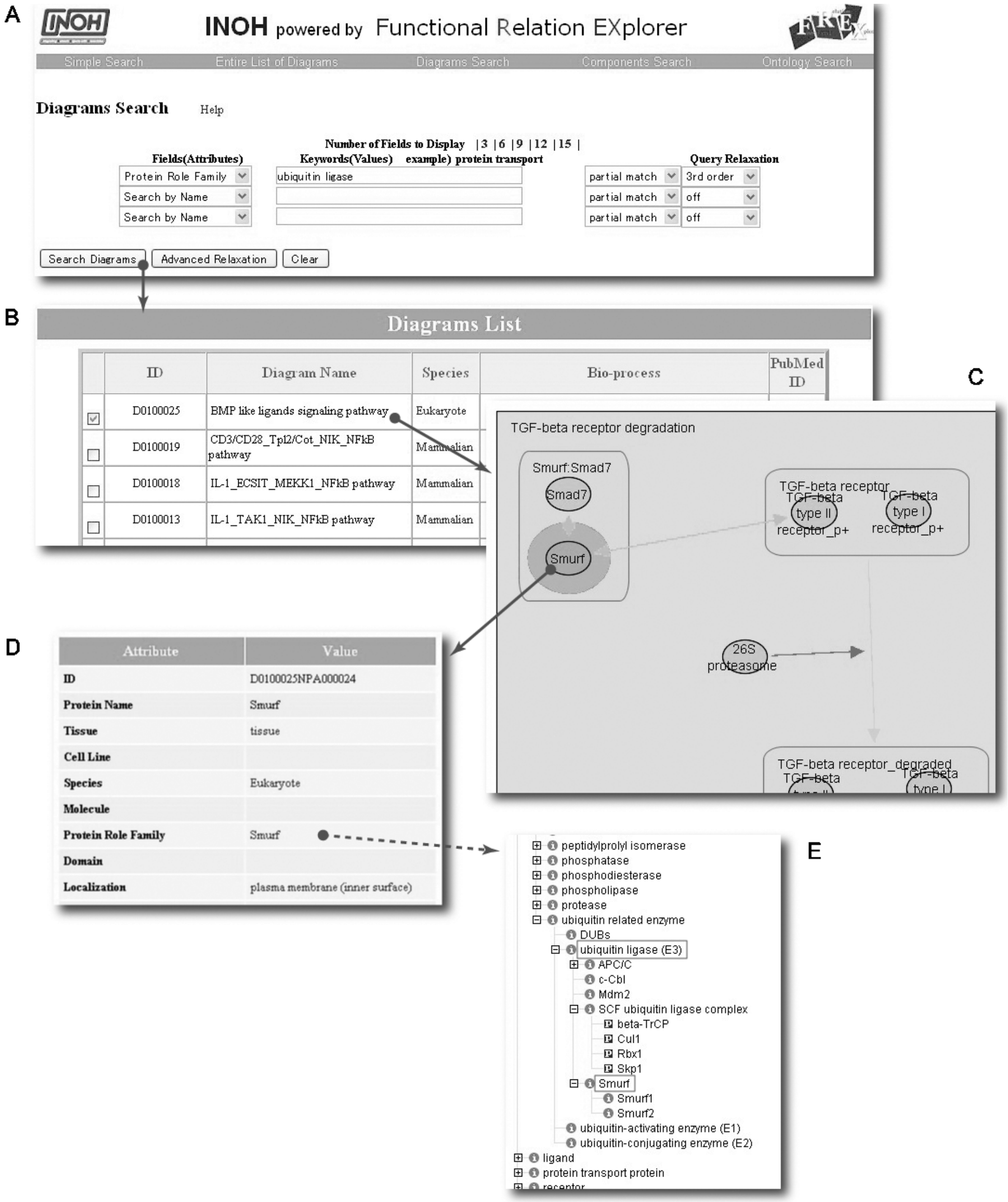

**Figure 4.** (A) FREX top page. To conduct a diagram search: (1) select an attribute type; (2) enter a keyword; (3) set a 'Query Relaxation' parameter. (B) A list of diagrams. (C) The highlight on the object indicates a hit. (D) Object attributes window. (E) Ontology display window information similar to what is represented by the Molecule Role Ontology's Function-Name nodes, Concrete-Name nodes (leaf nodes), and Complex-Names. Reactome defines the parent–children relation between these concepts by embedding a link into the attribute of each instance. This has some drawbacks, as discussed above. On the other hand, our system uses a set of ontologies (including the Molecule Role Ontology) that define relations between concepts and separate these from pathway objects.

### Future work

The Molecule Role Ontology has a small set of chemical substance terms. This is due to the fact that classifications of small-molecules such as IUPAC-IUBMB (IUPAC-IUBMB Joint Commission on Biochemical Nomenclature and Nomenclature Commission of IUBMB, 1992) or KEGG do not have terms like 'hormone' and 'second messengers'. On the other hand, our ontology does not have, for example, the terms Nucleic Acid, Lipid, and Amino Acid.

Synonyms for each term in the Molecule Role Ontology were extracted manually from the literature; as a result, its coverage of synonyms is low. We plan to incorporate gene symbols and synonyms from protein and gene databases by extracting these terms automatically. This would be done by following the Accession Nos of UniProt links.

The Molecule Role Ontology is by no means complete and we welcome any comments and feedback. The ontology is downloadable from our project URL (**http://www.inoh.org**).

### Conclusions

A resource like the Molecule Role Ontology greatly mitigates the burden of data curation from the scientific literature. By annotating molecule objects in pathway data with the Molecule Role Ontology, one can enrich the ambiguous descriptions of molecules in the literature with background knowledge, so that the system knows to which family the molecule belongs and how many sequence identities it may have. Additionally, using an ontology in a curation process reduces discrepancies in the data.

It also becomes easier to carry out the kind of complicated search that is difficult to achieve in a keyword-search-based database. First, it allows users to link related names. Although ERK1 cannot be found by a keyword-search that specifies MAPK as a query term, it can be found by an ontology-based search by carrying out a search over the children of MAPK on the Molecule Role Ontology. Some databases fulfil this function by defining keywords and synonyms relative to data on each molecule (the keyword 'MAPK' being attached to the 'ERK 1' molecule), but it is difficult to define a class relation, and this approach is inferior with regard to unitary management and the reusability of data. Second, it becomes possible to perform a query relaxation search that expands the concept relevant to the user-specified concept. By expanding the user-specified concept 'MAPK' according to the ontology (protein serine/threonine kinase), information about all molecules with the same function can be acquired.

### Acknowledgements

This work was supported in part by BIRD of the Japan Science and Technology Agency (JST), and by a Grant-in-Aid for Scientific Research on Priority Areas 'Genome Information Science' from the Ministry of Education, Culture, Sports, Science and Technology of Japan.

### References

AfCS website: **http://www.signaling-gateway.org/**

Apweiler R, Bairoch A, Wu CH, *et al*. 2004. UniProt: the universal protein knowledgebase. *Nucleic Acids Res* **32**: (Database Issue): D115–119.

Bader GD, Betel D, Hogue CW. 2003. BIND: the Biomolecular Interaction Network Database. *Nucleic Acids Res* **31**(1): 248–250.

BioCarta website: **http://www.biocarta.com/**

BioPAX website: **http://www.biopax.org/**

Boeckmann B, Bairoch A, Apweiler R, *et al*. 2003. The SWISS-PROT protein knowledgebase and its supplement TrEMBL in 2003. *Nucleic Acids Res* **31**(1): 365–370.

DAG-Edit website: **http://sourceforge.net/projects/geneontology**

Demir E, Babur O, Dogrusoz U, *et al*. 2004. An ontology for collaborative construction and analysis of cellular pathways. *Bioinformatics* **20**(3): 349–356.

Fukuda K, Takagi T. 2001. Knowledge representation of signal transduction pathways. *Bioinformatics* **17**(9): 829–837.

Fukuda K, Yamagata Y, Takagi T. 2003. FREX: a query interface for biological processes with a hierarchical and recursive structures. *In Silico Biol* **4**: 0007.

INOH database website: **http://www.inoh.org/**

Copyright © 2004 John Wiley & Sons, Ltd.

IUPAC-IUBMB Joint Commission on Biochemical Nomenclature and Nomenclature Commission of IUBMB. 1992. *Biochemical Nomenclature and Related Documents*, 2nd edn. Liébecq C (ed.). Portland Press: London.

Joshi-Tope G, Vastrik I, Gopinathrao G, *et al.* 2003. The Genome Knowledgebase: a resource for biologists and bioinformaticists. In *Cold Spring Harbor Symposia on Quantitative Biology*, vol LXVIII, Stillman B, Stewart D (eds). Cold Spring Harbor Laboratory Press: Cold Spring Harbor, New York; 237–244.

Kanehisa M, Goto S. 2000. KEGG: Kyoto Encyclopedia of Genes and Genomes. *Nucleic Acids Res* **28**(1): 27–30.

Karin M, Lin A. 2002. NF-$\kappa$B at the crossroads of life and death. *Nat Immunol* **3**(3): 221–227.

Karp PD, Arnaud M, Collado-Vides J, *et al.* 2004. The *E. coli* EcoCyc database: no longer just a metabolic pathway database. *ASM News* **70**(1): 25–30.

Krull M, Voss N, Choi C, *et al.* 2003. TRANSPATH®: an integrated database on signal transduction and a tool for array analysis. *Nucleic Acids Res* **31**(1): 97–100.

MeSH website: **http://www.nlm.nih.gov/mesh/meshhome.html**

Mulder NJ, Apweiler R, Attwood TK, *et al.* 2003. The InterPro Database 2003 brings increased coverage and new features. *Nucleic Acids Res* **31**(1): 315–318.

Salwinski L, Miller CS, Smith AJ, *et al.* 2004. The Database of Interacting Proteins: 2004 update. *Nucleic Acids Res* **32**: (Database Issue): D449–451.

Shields JM, Pruitt K, McFall A, Shaub A, Der CJ. 2000. Understanding Ras: 'it ain't over 'til it's over'. *Trends Cell Biol* **10**(4): 147–154.

STKE website: **http://stke.sciencemag.org/**

Takai-Igarashi T, Kaminuma T. 1999. A pathway finding system for the cell signaling networks database. *In Silico Biol* **1**: 0012.

The Gene Ontology Consortium. 2004. The Gene Ontology (GO) database and informatics resource. *Nucleic Acids Res* **32**: (Database Issue): D258–261.

Widmann C, Gibson S, Jarpe MB, Johnson GL. 1999. Mitogen-activated protein kinase: conservation of a three-kinase module from yeast to human. *Physiol Rev* **79**: 143–180.

Copyright © 2004 John Wiley & Sons, Ltd. 
